# Influence of environmental factors on *Cucumis melo* L. var. *agrestis* Naud. seed germination and seedling emergence

**DOI:** 10.1371/journal.pone.0178638

**Published:** 2017-06-01

**Authors:** Hongle Xu, Wangcang Su, Di Zhang, Lanlan Sun, Hengliang Wang, Fei Xue, Shunguo Zhai, Zeguo Zou, Renhai Wu

**Affiliations:** 1 Institute of Plant Protection, Henan Academy of Agricultural Sciences, Zhengzhou, P.R. China; 2 Henan Key Laboratory of Crop Pest Control, Zhengzhou, P.R. China; 3 Key Laboratory of Integrated Pest Management on Crops in Southern Region of North China, Zhengzhou, P.R. China; 4 Luoshan Institute of Agricultural Sciences, Xinyang, P.R. China; California State University Fresno, UNITED STATES

## Abstract

*Cucumis melo* L. var. *agrestis* Naud. (field muskmelon) is an annual invasive weed in many parts of Asia. However, there is very little available information about the germination and emergence of this species. Therefore, laboratory experiments were conducted to evaluate the effects of light, temperature, salt stress, osmotic stress, pH, and depth of planting on field muskmelon germination and seedling emergence. Light had no effect on seed germination, and the seeds germinated at a wide range of temperatures. More than 90% of the seeds germinated at constant temperatures between 20°C and 35°C, and fluctuating day/night temperatures between 15/25 and 30/40°C. The seeds were tolerant to salinity as germination occurred up to the 200 mM NaCl treatment. However, the seeds were sensitive to osmotic stress as seed germination was completely inhibited at –0.6 MPa. The seeds germinated over a pH range of 4 to 10, which suggested that pH was not a limiting factor for germination. Seedling emergence was greatest (97.86%) when the seeds were planted on the soil surface, but emergence declined as the burial depth increased. Information from this study can be used to predict future infestations in China and help develop strategies to manage this species.

## Introduction

*Cucumis melo* L. var. *agrestis* Naud. (field muskmelon) is an annual, monoecious, trailing-vine weed that belongs to the *Cucurbitaceae* family. It is native to Africa and has invaded Shandong, Jiangsu, Anhui, Henan, and Shanghai, China [1]. This species is a common intruder in natural open areas, slopes, grasslands, wastelands, and fields. It can also infest crops, such as soybean, peanuts, and corn [2–4]. The main differences between this weed melon and cultivated melon are the sizes of the plant and the fruit. Field muskmelon is more slender than the cultivated species and its fruits are ellipsoid, sweet, sour, or bitter, with dimensions ranging from 2 to 6 cm in diameter and 2 to 10 cm in length [5].

During the 1960s and 1970s, a comprehensive investigation of potentially useful wild plants was conducted and field muskmelon cultivation began as an oilseed-crop in Fuyang City, Anhui Province, China [5]. This species is used for making soy sauce and vinegar. The flesh of the melon is used for wine, and melon vines are used as raw material for paper and artificial cotton [5]. Field muskmelon has been partially successful as a crop, but its cultivation has led to serious infestations as a weed in many places. Based on our survey, there are more than 10 fruits per field muskmelon plant and this weed is easily dispersed after fruit maturity. It has a tendril that can be used to entwine itself around other plants and reduce photosynthesis. For example, it decreased corn grain yield by 20%–50% [6].

To prevent economic and ecological diversity losses, the basic biology and ecology of a weed species should be investigated before it is introduced into new areas [7, 8]. The rapid propagation of invasive plants is usually correlated with a high seed germination percentage. Seed germination and the emergence of a weed species are influenced by environmental factors, such as light, temperature, salt, osmotic soil salinity, soil solution osmotic potential, pH, and soil burial depth [9–11].

Many plants germinate when the seeds are on or near the soil surface as they require light for germination [12]. Thus, their germination can be inhibited when buried deeper. Temperature is a major environmental factor that influences seed germination and each species has an optimal range for germination [13]. Temperature can regulate enzyme activities, and promote or inhibit the synthesis of hormones that affect seed germination [14]. Additionally, salinity stress and the osmotic potential of the soil and/or water are other important factors that determine seed germination. Previous research has shown that field muskmelon could not survive in soils with a low osmotic potential [15] compared to other species like *Euphorbia heterophylla* which germinate even at a soil moisture tension of –0.8 MPa [16]. Soil pH also has a great impact on weed seed germination. The factors determining soil pH are parent material, precipitation, vegetation, soil amendments, and organic matter levels. Furthermore, germination is affected if inorganic matter level in the soil is more than 90% [17].

Another factor influencing germination and seedling emergence is seed burial depth because it is influenced by moisture availability, soil temperature, and light exposure at different depths. Furthermore, burial depth also has a significantly large effect on weed control [9].

A better understanding of the effects of environmental factors on field muskmelon seed germination and emergence may help to predict the areas where it may potentially invade and provide information on effective control measures. To date, very little available information is available on the germination and emergence of this weedy species. Therefore, the objectives of this study were to determine the effects of light, temperature, salt stress, osmotic stress, pH, and burial depth on field muskmelon germination and seedling emergence.

## Materials and methods

### Seed source

The field muskmelon seeds were collected during October, 2015 from soybean fields at the Henan Academy of Agricultural Sciences in Xinxiang (N 35°18', E 113°52'), Henan, China. Mature seeds were collected from at least 100 plants. The seeds were cleaned and stored in paper bags at room temperature (20 ± 5°C) until further use.

This study did not involve endangered or protected species. No specific permissions were required for the location where the field muskmelon seeds were collected.

### General germination test protocol

Unless stated otherwise, germination was determined in 9 cm diameter Petri dishes (Wuyi Glass Instrument Manufacture, Shanghai, P.R. China) lined with two pieces of Whatman No.1 filter paper (GE Healthcare Bio-Sciences, PA, USA). Each Petri dish was soaked with 5 ml of distilled water (pH = 6.8) or the test solutions. Twenty seeds were placed in each dish. All the seeds were treated with 5% NaClO for 5 min and then washed with running tap water. Parafilm was used to seal the Petri dishes to reduce water evaporation. The Petri dishes were placed in incubators (GXZ-300B, Jiangnan Instrument Manufacture, Ningbo, China) with fluctuating day/night temperatures of 30/20°C under 12 h light/12 h dark conditions unless otherwise specified. Fluorescent lamps were used to produce a photosynthetic photo flux density of 88 μM m^−2^ s^−1^. The seeds were considered to have germinated when the radicles were visible (> 2 mm) and germinated seeds were removed after every count. Non-germinated seeds were tested with a 1% TTC (2, 3, 5-triphenyltetrazolium chloride) at 30°C to assess viability. After 12 h, seeds showing a pink to reddish colored were considered viable [18].

### Dormancy

Seeds were collected at maturity and stored in paper bags at room temperature (20 ± 5°C). They were then used to determine whether field muskmelon seed has dormancy characteristics or not. Seed germination tests were performed at 2, 6, and 10 days (d) after collection and each treatment contained 20 seeds. The other environmental conditions were the same as those described in the general germination test protocol. Seed germination was recorded daily until 12 d after sowing.

### Effect of light on germination

The effect of light on germination was studied by incubating the seeds in continuous (24 h) light, continuous (24 h) dark, and alternating light/dark (12 h/12 h) regimens. The dark treatment involved wrapping the dish in two layers of aluminum foil immediately after sowing, but the light only treatment dishes were continuously exposed to light. A fluctuating day/night temperature of 30/20°C was maintained for all the tests. The other environmental conditions were the same as those described in the general germination test protocol. Seed germination was recorded daily until 28 d after sowing for the light/dark and continuous light regimes, and only after 28 d for those grown under dark conditions.

### Effect of temperature on germination

To determine the effects of temperature on germination, the seeds were placed in incubators under constant (15, 20, 25, 30, or 35°C) or fluctuating temperatures (day/night temperatures: 40/30, 35/25, 30/20, 25/15, or 20/10°C) under light/dark (12/12 h) conditions. The other environmental conditions were same as those described in the general germination test protocol. Seed germination was recorded daily until 28 d after sowing.

### Effect of salt stress on germination

The influence of salinity on field muskmelon germination was determined by placing the seeds in sealed dishes containing 5 ml of the different concentrations (0, 25, 50, 100, 150, 200, 250, 300, 350 or 400 mM) of sodium chloride (NaCl). Other conditions were the same as the general germination test protocol mentioned previously. Seed germination was recorded daily until 34 d after sowing.

### Effect of osmotic stress on germination

The effect of osmotic stress on germination was determined by placing seeds in solutions with water potentials of 0, –0.1, –0.2, –0.4, –0.6, –0.8, –1.0, –1.2, –1.4, or –1.6 MPa, which were adjusted by dissolving 0, 79.55, 118.94, 175.00, 218.14, 254.55, 286.65, 315.67, 342.37, or 367.23 g of polyethylene glycol (PEG) 8000 in 1 L distilled water [19]. The other environmental conditions were the same as those described in the general germination test protocol. Seed germination was recorded daily until 28 d after sowing.

### Effect of pH on germination

The effect of pH on germination was determined by placing seeds in buffer solutions with pH values of 4, 5, 6, 7, 8, 9, or 10, or in distilled water (pH 6.8) as the control. The pH 4 buffer solution was made up with 2 mM adjacent benzene dicarboxylic acid potassium hydrogen and 1 M HCl. Then, 2 mM 2- (*N*- morpholino) ethane sulfonic acid (MES), and 1 M NaOH were used to adjust the solution to pH 5 or 6. Similarly, 2 mM 4 hydroxyethyl piperazine ethyl sulfonic acid (HEPES) and 1 M NaOH were used to adjust the solution to pH 7 or 8. The pH 9 and 10 buffers were prepared using 2 mM three (hydroxymethyl) *N*- methyl glycine (tricine) and 1 M NaOH. The other environmental conditions were the same as those described in the general germination test protocol. Seed germination was recorded daily until 21 d after sowing.

### Effect of burial depth on seedling emergence

The effect of burial depth on seedling emergence was determined using 8 cm diameter, 16 cm high plastic pots in incubators. Twenty seeds were sown in each pot at either 0 cm depth (soil surface) or covered with soil (a 2:1 wt/wt mixture of sand and soil, pH 5.6, 1.4% organic matter) to a depth of 1, 2, 4, 6, or 8 cm. The soil was collected from an uncultivated area that had never been treated with herbicide and was then passed through a 2 mm sieve. The pots were placed randomly inside the incubator and their positions were changed every day. The other environmental conditions were the same as those described in the general germination test protocol. The pots were watered as needed to maintain optimal moisture levels. Seedling emergence was defined as when the coleoptile could be visibly distinguished, and seed emergence was counted daily until 30 d after sowing. Finally, the non-emerged seeds were checked to determine whether seeds failed to germinate or whether the coleoptile had failed to break through the soil surface. Additionally, non-seeded pots were used as controls to determine the seed bank in the soil. No field muskmelon was found in these control pots, which suggested that there was no background field muskmelon seed bank in the soil.

### Statistical analysis

All the experiments were carried out in a randomized complete block design and each treatment was replicated four times. Each experiment was repeated twice and the data were subjected to ANOVA. The data were pooled for analysis as there was no significant (α > 0.05) trial-by-treatment interaction.

Germination data from the temperature, pH, salt, and osmotic stress studies were best fitted to a functional three-parameter sigmoid model using SigmaPlot 10.0 (Systat software Inc., San Jose, CA, USA) as shown in Equation 1:

$$\text{G}={\text{G}}_{\text{max}}/\left\{1+\text{exp}\left[-\left(\text{x}-{\text{T}}_{50}\right)/{\text{G}}_{\text{rate}}\right]\right\}$$

where G is the total germination (%) at time x and was calculated using the number of viable seeds (tested by TTC), G_max_ is the maximum germination (%), T_50_ is the time taken to reach 50% of the final germination, and G_rate_ indicates the slope of T_50_.

The seedling emergence data at different burial depths were fitted to a functional three-parameter sigmoid model using SigmaPlot 10.0 as shown in Equation 2:

$$\text{E}={\text{E}}_{\text{max}}/\left\{1+\text{exp}\left[-\left(\text{x}-{\text{T}}_{50}\right)/{\text{E}}_{\text{rate}}\right]\right\}$$

where E is the total seedling emergence (%) at burial depth x and was calculated using the number of viable seeds (tested by TTC), E_max_ is the maximum seedling emergence (%), T_50_ is the time taken to reach 50% of the final emergence, and E_rate_ indicates the slope of T_50_.

Significant differences for G_max_ or E_max_ among treatments were identified using Tukey’s test (P < 0.05) after analysis by SPSS 18.0 software (SPSS Inc., Chicage, IL).

## Results and discussion

### Field muskmelon dormancy

**Fig 1:**
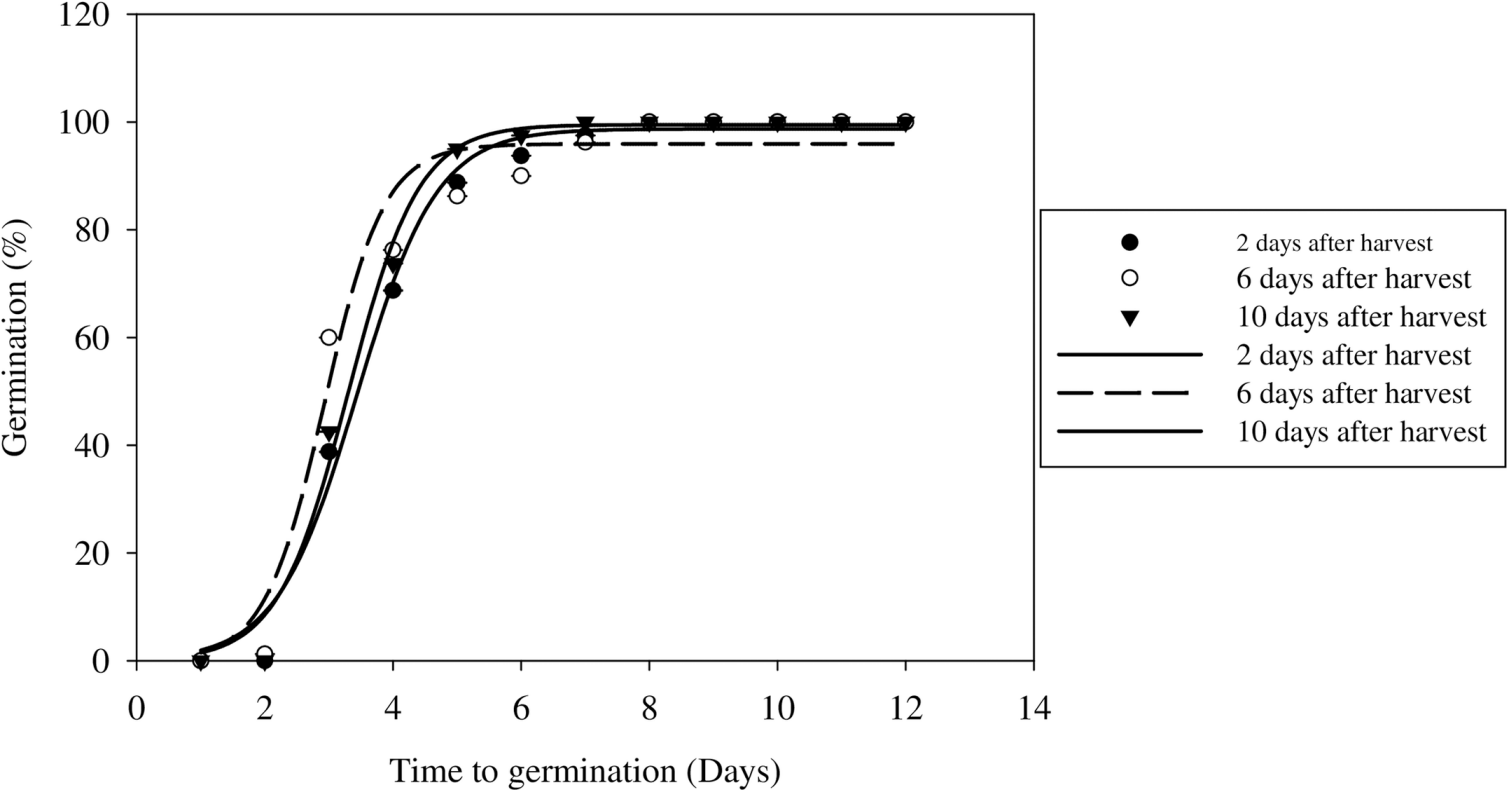
Field muskmelon germination at 2, 6, and 10 d after harvest. Error bars represent standard errors of the means. Values represent the mean of eight replications with 20 seeds per replication.

Field muskmelon seeds began to germinate 2 d after sowing and germination increased over time. All the seeds germinated after 8 d (Fig 1). There were no significant differences in seed germination when they were sown at different times after harvest, and the G_max_ values were more than 95% (Table 1). The T_50_ values were about 3 d for the different sowing times after harvest (Table 1). These results showed that field muskmelon has no innate dormancy.

**Table 1:** Field muskmelon germination after harvest.

Day (d)	G_max_^a^ (S.E.)^b^	G_rate_	T_50_	R^2^
2	98.72 a (1.56)	0.6248	3.44	0.9911
6	95.92 a (2.62)	0.4674	2.93	0.9680
10	99.51 a (1.42)	0.5503	3.30	0.9923

^a^ Means within a column followed by different letters indicate significant differences (P < 0.05) according to Tukey’s test.

^b^ standard error of the mean.

Dormancy is an internal state whereby seed germination is inhibited at certain moisture, temperature, and oxygen levels [20]. Seed dormancy has an important ecological significance and can effectively regulate the temporal and spatial distribution of seed germination. It is an effective strategy that is used to resist environmental instability [21]. In this study, field muskmelon germination increased significantly at only 2 d after harvesting, which indicated that this weed could germinate immediately after maturity when the environmental conditions are suitable. This result is in contrast to other related weed species, such as *C*. *melo* L. subsp. *agrestis* var. *agrestis* (Naudin) Pangalo [22].

### Effect of light on germination

**Fig 2:**
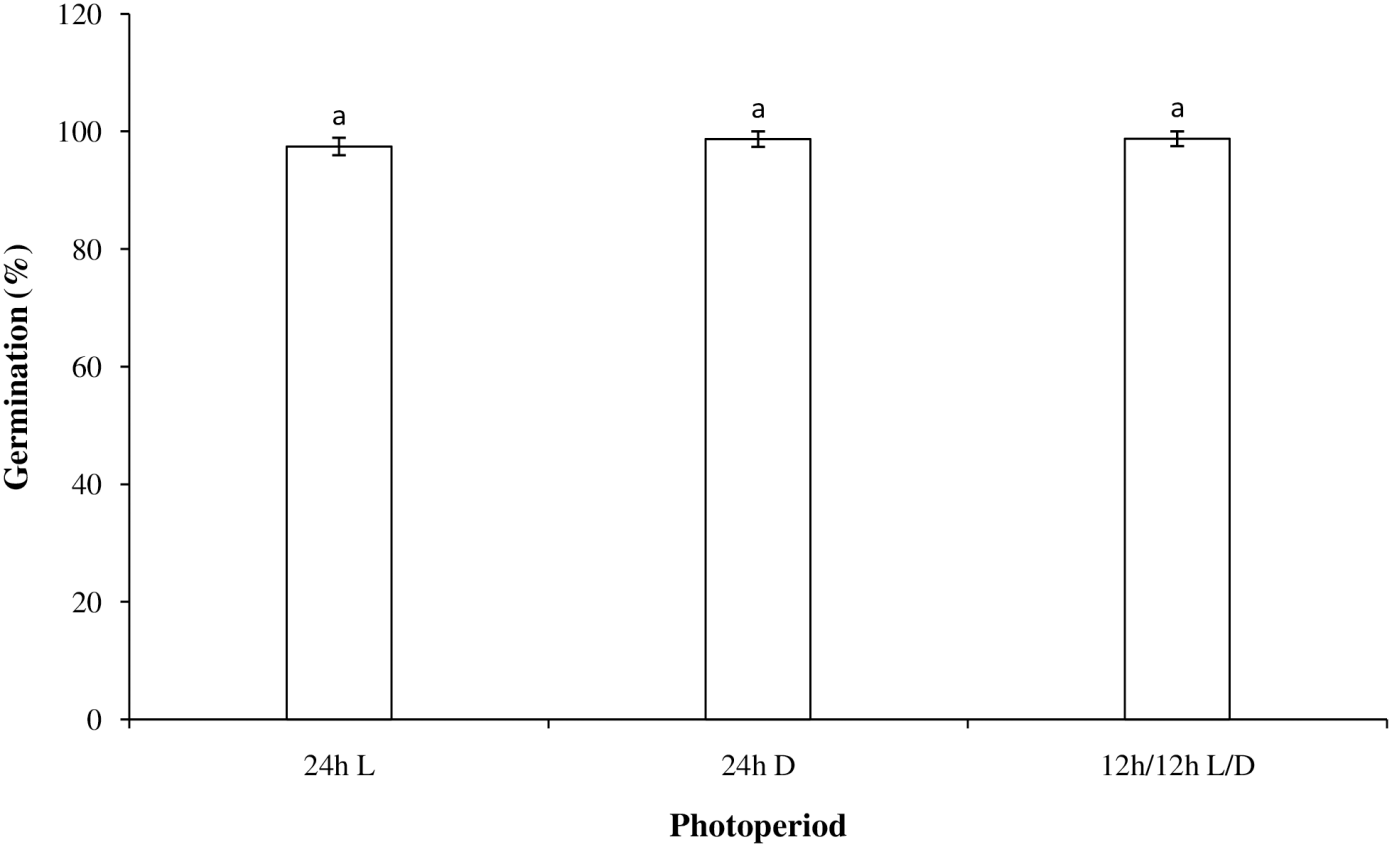
Effect of light on the germination of field muskmelon incubated at 30/20°C. Error bars represent standard errors of the means. Values represent the mean of eight replications with 20 seeds per replication. Different letters indicate significant differences (P <0.05) according to Tukey’s test.

There were no significant differences in field muskmelon seed germination between the light, dark, and light/dark treatments (Fig 2). Germination was above 97% for all treatments, which indicated that light was not a necessary factor for field muskmelon germination. Similar results have been observed for other weeds, such as *Citrullus lanatus* Thunb. [23], *Sida spinosa* L. [24] and *Poa annua* L. [25]. Field muskmelon’s insensitivity to light during germination partly explains why this weed has become problematic in both no-till and shallow tillage farming systems.

### Effect of temperature on germination

Seed germination was significantly affected by constant temperature and fluctuating temperatures (P < 0.05), which suggested that temperature has an important influence on seed germination like in most plant species.

**Fig 3:**
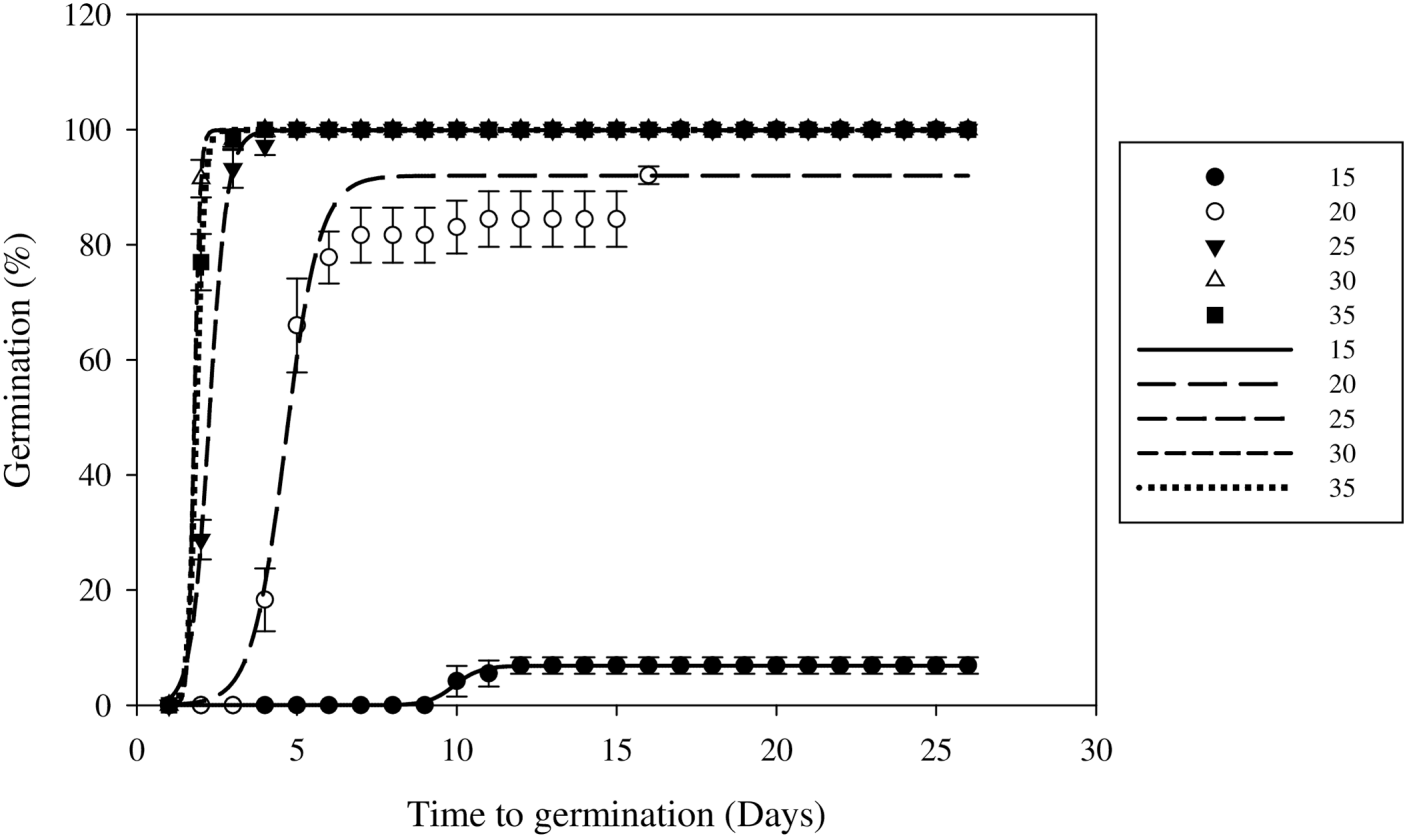
Effect of constant temperature on field muskmelon germination after incubation under a 12 h photoperiod regime. Error bars represent standard errors of the means. Values represent the mean of eight replications with 20 seeds per replication.

At the constant temperatures used in this study, germination slightly increased as the temperature was increased (Fig 3). Field muskmelon seeds germinated at all constant temperatures, and the G_max_ values were similar at temperatures above 20°C (Table 2). Maximum germination (99.96%) occurred at 35°C and minimum germination (7.99%) occurred at 15°C. T_50_ decreased from 10.1 to 1.9 d, as the temperature was increased from 15 to 35°C (Table 2). It took longer for the seeds to begin to germinate as the temperature decreased (Fig 3).

**Table 2:** Effect of constant temperature (°C) on field muskmelon seed germination.

Temperature (°C)	G_max_^a^ (S.E.)^b^	G_rate_	T_50_	R^2^
15	6.99 b (0.06)	0.5581	10.10	0.9961
20	91.91 a (1.75)	0.5234	4.65	0.9736
25	99.88 a (0.12)	0.2849	2.26	0.9997
30	99.92 a (0.07)	0.0916	1.79	0.9998
35	99.96 a (0.06)	0.1207	1.85	1.0000

^a^ Means within a column followed by different letters indicate significant differences (P < 0.05) according to Tukey’s test.

^b^ standard error of the mean.

**Fig 4:**
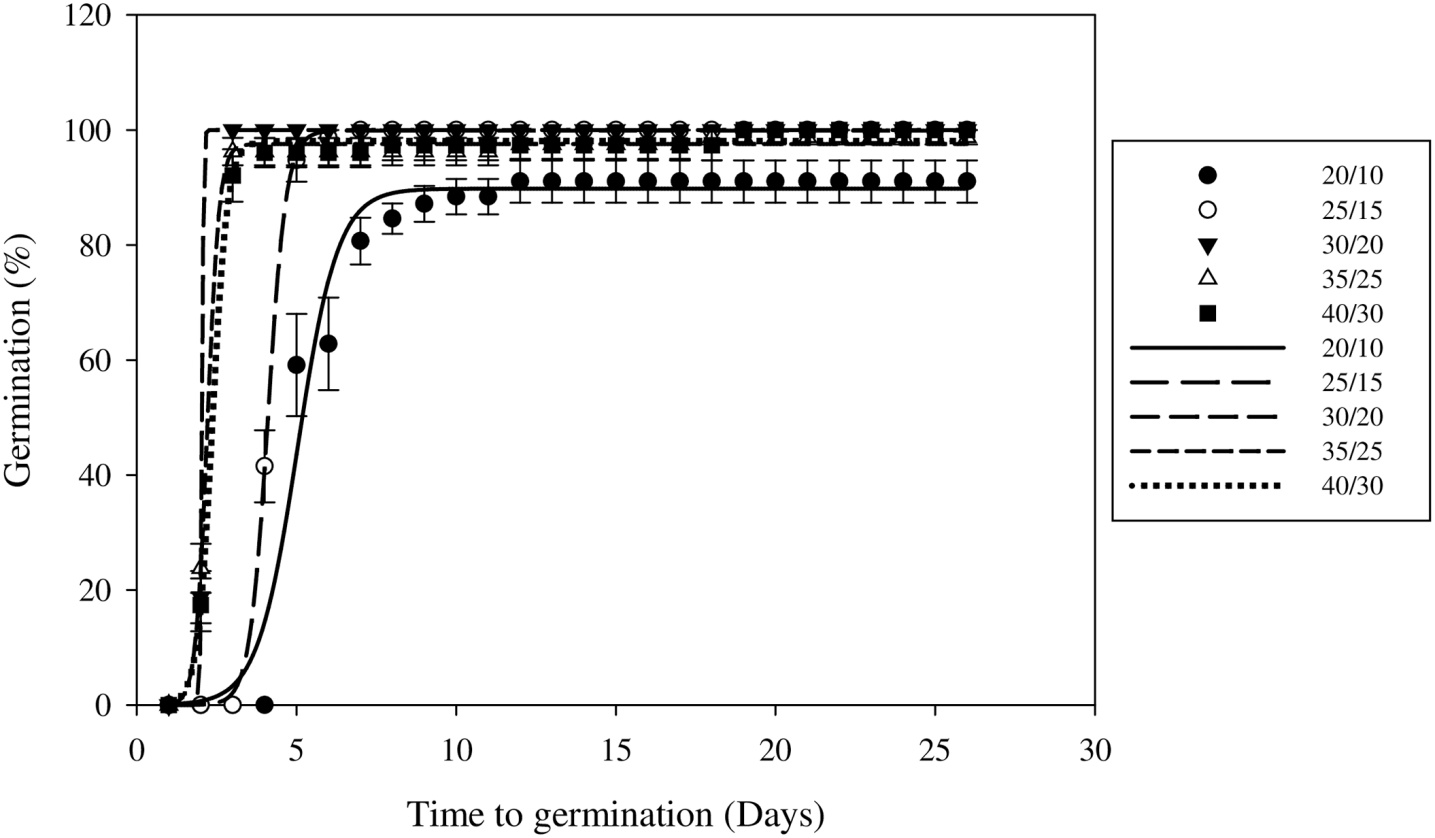
Effect of fluctuating temperature on field muskmelon germination after incubation under a 12 h photoperiod regime. Error bars represent standard errors of the means. Values represent the mean of eight replications with 20 seeds per replication.

Under the fluctuating temperature regimes, the G_max_ values of the different treatments were all more than 85% (Fig 4). The highest seed germination (100%) was observed at 30/20°C and this rate was greater than the germination observed under the 20/10°C fluctuating temperature regime. The G_max_ value was lower under the 35/25°C (97.55%) and 40/30°C (98.06%) treatments. Field muskmelon under the 30/20°C fluctuating temperature treatment had the lowest T_50_ and highest G_max_ value, which indicates that 30/20°C was the most suitable fluctuating temperature for germination (Table 3).

**Table 3:** Effect of fluctuating temperature (°C) on field muskmelon seed germination.

Temperature (°C)	G_max_^a^ (S.E.)^b^	G_rate_	T_50_	R^2^
20/10	89.98 b (1.23)	0.6269	5.04	0.9878
25/15	99.9 a (0.12)	0.2865	4.10	0.9999
30/20	100 a (0.00)	0.0298	2.04	1.0000
35/25	97.55 a (0.22)	0.1839	2.21	0.9900
40/30	98.06 a (0.31)	0.2345	2.36	0.9983

^a^ Means within a column followed by different letters indicate significant differences (P < 0.05) according to Tukey’s test.

^b^ standard error of the mean.

In this study, constant temperatures of 20/35°C and the fluctuating 15/25 to 30/40°C regimes were all suitable for field muskmelon germination. Based on our observations, field muskmelon germinates from late May to October in Henan Province, China, when the temperature is most suitable for field muskmelon germination. The lower germination (6.99%) at constant temperatures of 15°C suggested that this weed might be less problematic in cooler or higher altitude areas. The temperature used in this study indicated that field muskmelon seeds need warm environmental conditions for germination. These results were similar to other summer broadleaf weed species, such as *Bidens pilosa* [26], *Eclipta prostrate* [27], and *Chamaesyce maculate* [28]. Edelstein *et al*. [29] reported that seeds of the *Cucurbitaceae* family require warm temperatures to germinate, and low temperatures had an effect on germination. The temperature data for field muskmelon seed germination can help inform phenological studies and new strategies for managing this summer weed species.

### Effect of salt stress on germination

**Fig 5:**
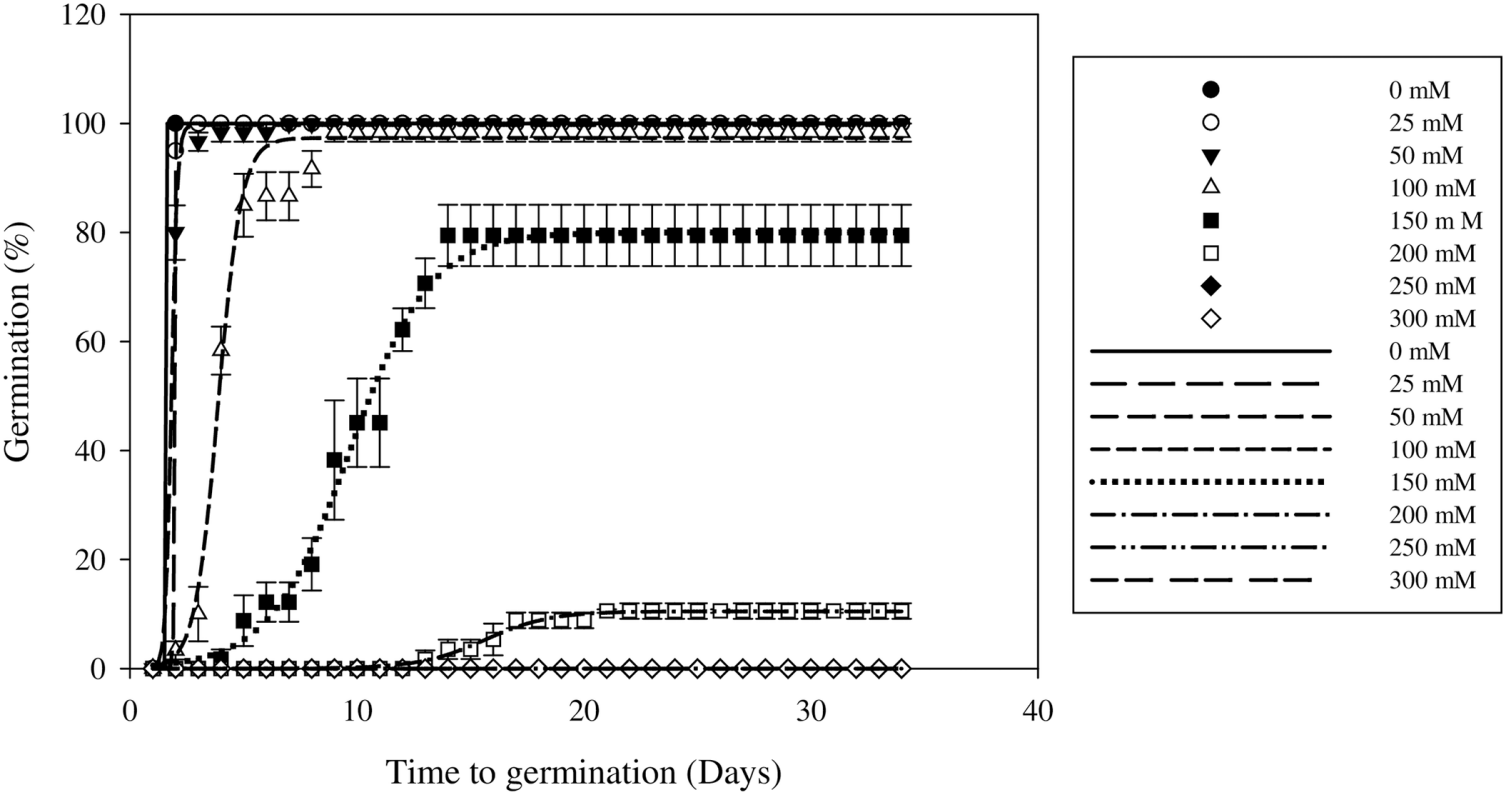
Effect of salt stress (mM) on field muskmelon germination after incubation at 30/20°C under a light/dark regime. Error bars represent standard errors of the means. Values represent the mean of eight replications with 20 seeds per replication.

Salt stress significantly delayed the start of germination and reduced the germination percentage of the seeds (Table 4 and Fig 5). Seed germination decreased slightly as the salt concentration increased from 0 to 100 mM, but declined sharply at 150 to 300 mM (Fig 5). Germination was more than 90% up to a concentration of 100 mM NaCl. The seeds had the highest cumulative germination (100%) in the control treatment and at 25 mM NaCl. Field muskmelon germination was 80% at 150 mM NaCl, but decreased considerably to 10% at 200 mM NaCl. No germination was observed at NaCl concentrations of 250 and 300 mM. The T_50_ values increased as the salt concentration rose (Table 4).

**Table 4:** Effect of salt stress (mM) on field muskmelon seed germination.

NaCl (mM)	G_max_^a^ (S.E.)^b^	G_rate_	T_50_	R^2^
0	100.00 a (0.00)	0.0029	1.57	1.0000
25	100.00 a (0.00)	0.0117	1.98	1.0000
50	99.74 a (0.13)	0.1303	1.82	0.9980
100	97.34 a (0.58)	0.5139	3.88	0.9880
150	80.02 b (0.62)	1.7661	9.69	0.9930
200	10.50 c (0.12)	1.4466	15.63	0.9920
250	0 d (0.00)	0	0	0
300	0 d (0.00)	0	0	0

^a^ Means within a column followed by different letters indicate significant differences (P < 0.05) according to Tukey’s test.

^b^ standard error of the mean.

Soils containing more than 100 mM NaCl are considered to be highly saline [30]. In this study, the germination percentage for field muskmelon was 80% at 150 mM NaCl, which showed that this weed has a strong tolerance to salinity. These results suggest that field muskmelon may be able to colonize saline areas because some seeds could still germinate at 200 mM NaCl. Similar results have been reported for *C*. *melo* L., where seeds germinated at 150 mM NaCl. This demonstrated that the seeds had a high genetic potential for salt tolerance [31]. However, these results were in contrast with Tanveer *et al*. [32], who reported that *C*. *melo* var. *agrestis*.seed germination was low (15%) at 150 mM NaCl. This may be explained by the different temperatures used in the salt stress experiment (30/20°C) during this study. They were also different from the temperatures used by Sohrabikertabad *et al*. (38/35°C) [3], who also reported that different temperatures had an effect on germination at certain salt stress levels. Also, it is not known if the seedlings can establish and grow under saline conditions. Studies need to be conducted to quantify this.

### Effect of osmotic stress on germination

**Fig 6:**
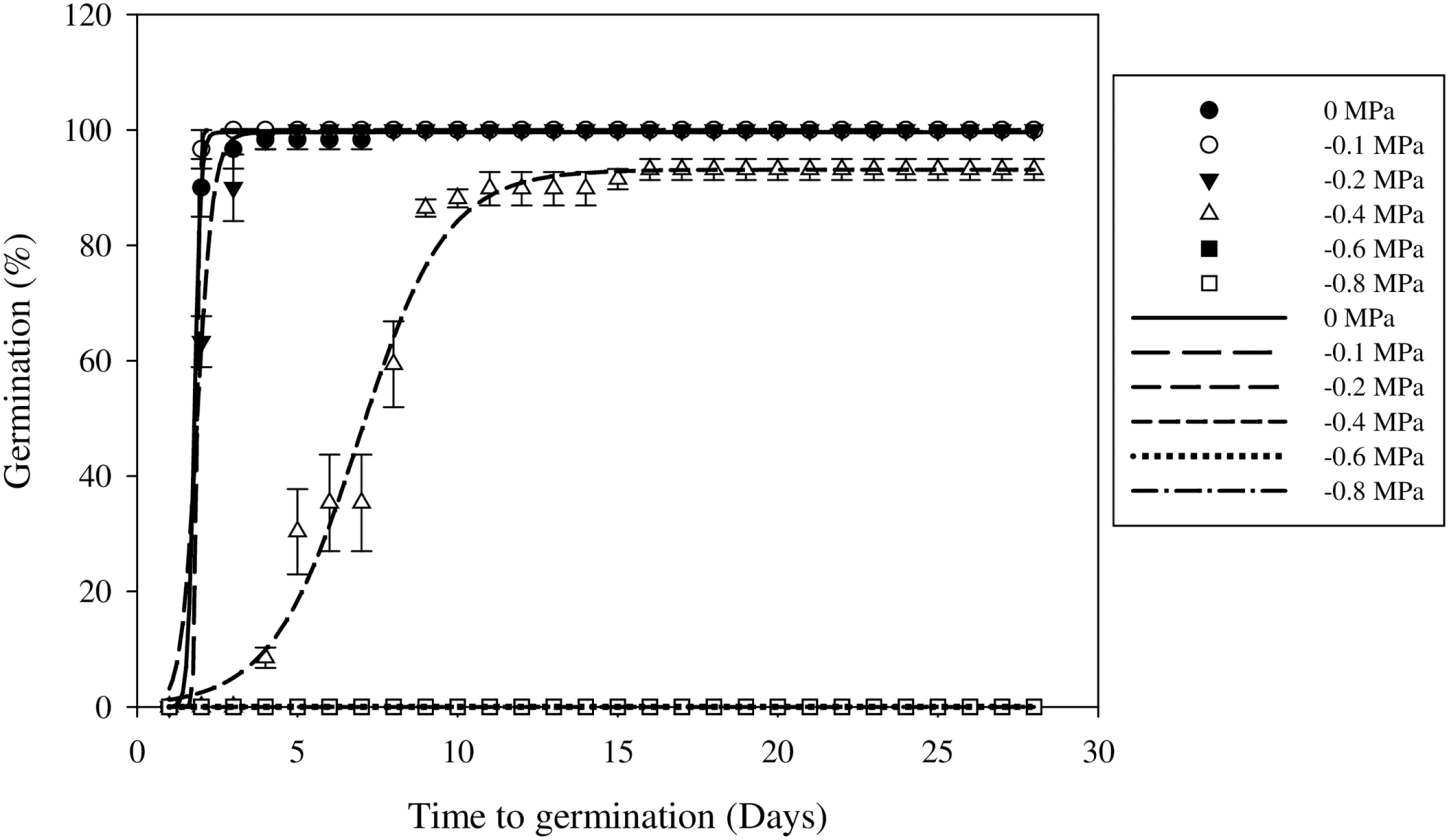
Effect of osmotic stress on field muskmelon germination after incubation at 30/20°C under a light/dark regime. Error bars represent standard errors of the means. Values represent the mean of eight replications with 20 seeds per replication.

Seed germination decreased as osmotic stress increased (Fig 6). There were no significant differences in seed germination up to an osmotic stress of –0.4 MPa and the germination rates were > 90% (Table 5). At an osmotic potential of –0.4 MPa, seed germination was 93%, but the T_50_ was 6.9 d, which was about 3.8 times longer than the control (Table 5). Germination was entirely inhibited at –0.6 MPa, which suggested that field muskmelon seeds favor moist environments, whereas seeds under drought conditions may not germinate until the conditions were favorable. This ability also suggests that the seeds will not germinate until after a rainfall event, which prolongs the existence of these seeds in the seed bank.

**Table 5:** Effect of osmotic stress on field muskmelon seed germination.

osmotic stress (MPa)	G_max_^a^ (S.E.)^b^	G_rate_	T_50_	R^2^
0	99.62 a (0.17)	0.0987	1.78	0.9981
–0.1	100.00 a (0.03)	0.0418	1.86	1.0000
–0.2	99.62 a (0.37)	0.2532	1.87	0.9918
–0.4	93.11 a (1.11)	1.3699	6.92	0.9842
–0.6	0 b (0.00)	0	0	0
–0.8	0 b (0.00)	0	0	0

^a^ Means within a column followed by different letters indicate significant differences (P < 0.05) according to Tukey’s test.

^b^ standard error of the mean.

The results suggested that field muskmelon seeds were sensitive to water deficit and that this was consistent with its growing season, where its germination and growing periods occur in the summer months (rainy season). Summer rainfall across China can account for 30%–60% of the annual total precipitation [33]. The moist soils in summer enable field muskmelon to germinate, which can partly explain why it occurs throughout China, and endangers corn, soybean, peanut, and other summer crops. Similar results were recorded by Ramirez *et al*. for *Citrullus lanatus* var. *citroides* [34]. The germination rate declined to 20% at osmotic potential of –0.6 MPa and no germination was occurred at > –0.9 MPa [34]. *Ipomoea triloba* germination is also sensitive to water availability, and its seeds did not germinate at a water potential of –0.6 MPa or below [35]. Awan *et al*. [30] also reported that *Urena lobata* L. seed germination decreased linearly when the osmotic potential fell from 0 to –0.8 MPa.

### Effect of pH on germination

**Fig 7:**
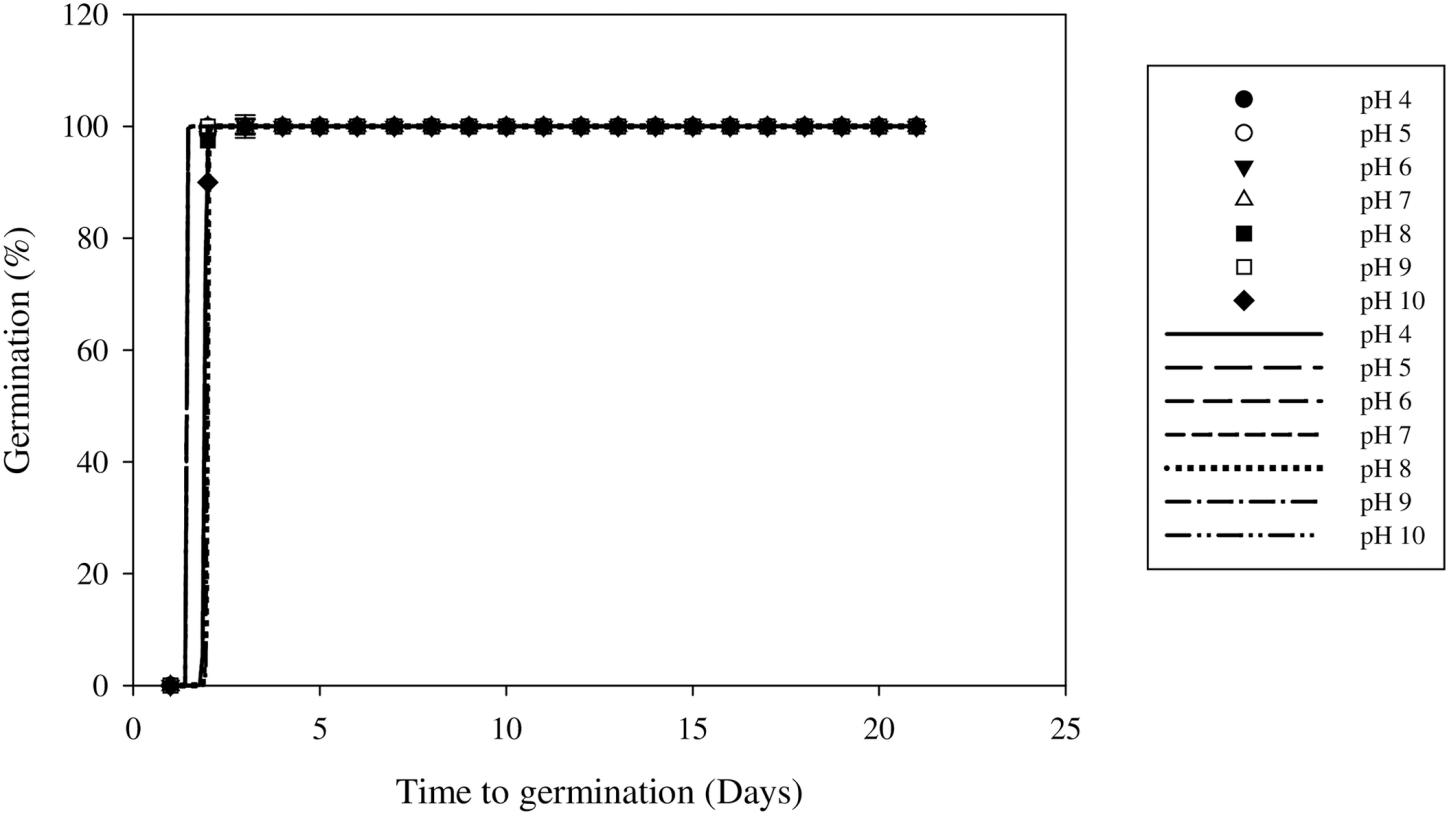
Effect of pH on field muskmelon germination after incubation at 30/20°C under a light/dark regime. Error bars represent standard errors of the means. Values represent the mean of eight replications with 20 seeds per replication.

Seed germination was not affected by pH (P < 0.05) (Table 6). Germination began immediately and all the seeds had germinated within 48 hours. No significant differences were detected over the pH range used in this study, and the germination rates of all treatments were greater than 90% (Table 6 and Fig 7).

**Table 6:** Effect of pH on field muskmelon seed germination.

pH	G_max_^a^ (S.E.)^b^	G_rate_	T_50_	R^2^
4	100.00 a (0.00)	0.0194	1.92	1.0000
5	100.00 a (0.00)	0.0008	1.46	1.0000
6	100.00 a (0.00)	0.0194	1.92	1.0000
7	100.00 a (0.00)	0.0008	1.46	1.0000
8	99.88 a (0.01)	0.0017	1.96	0.9994
9	100.00 a (0.00)	0.0008	1.46	1.0000
10	100.00 a (0.00)	0.0104	1.98	1.0000

^a^ Means within a column followed by different letters indicate significant differences (P < 0.05) according to Tukey’s test.

^b^ standard error of the mean.

The ability of field muskmelon to germinate over a wide range of pHs indicates that pH value is not a major limiting factor on this weed. In China, the soil pH range is wide, and most of the soil pH values are between 4.5 and 9 [36]. Field muskmelon is well adapted to this pH range. The results of this study were similar to the results for *E*. *heterophylla* seeds, which have also been reported to germinate over a wide range of pH values [16].

### Effect of burial depth on seedling emergence

**Fig 8:**
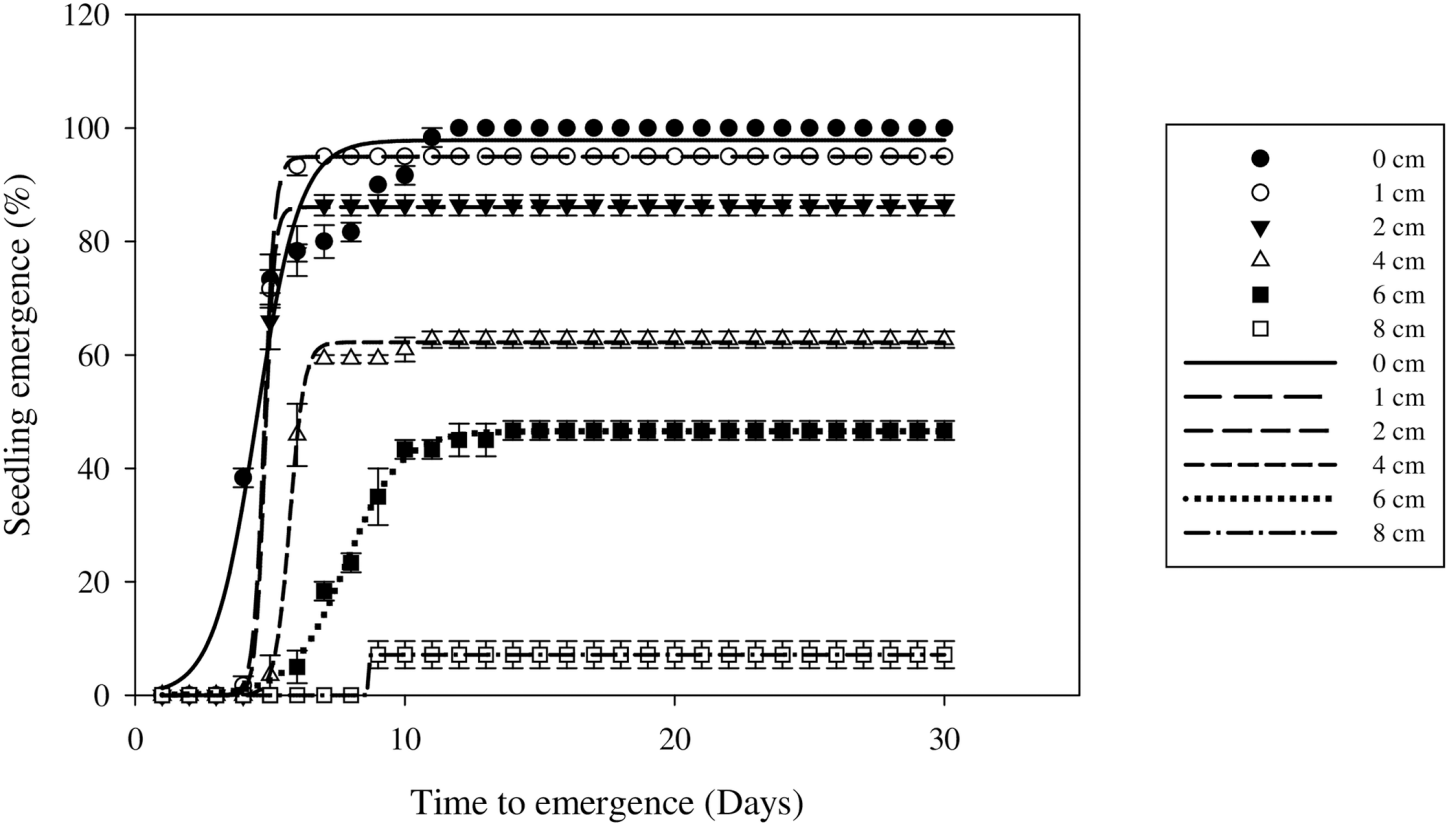
Effect of burial depth on field muskmelon germination after incubation at 30/20°C under a light/dark regime. Error bars represent standard errors of the means. Values represent the mean of eight replications with 20 seeds per replication.

The emergence of field muskmelon seedlings was significantly affected by burial depth (P < 0.05). Seedling emergence decreased rapidly as the burial depth increased (Fig 8). Seeds placed on the soil surface and at a seed burial depth of 1 cm showed similar emergence rates of more than 90% (Table 7). Emergence steadily decreased as the burial depth increased beyond 1 cm. Seedling emergence reached a maximum (97.86%) when the seeds were placed on the soil surface, whereas only 7.14% emerged when the seeds were buried at a depth of 8 cm. Increasing the burial depth led to a rise in the T_50_ values. The T_50_ values were 5.7, 7.8, and 8.7 d at seed burial depths of 4, 6, and 8 cm, respectively (Table 7).

**Table 7:** Effect of burial depth on field muskmelon seed germination.

Burial depth (cm)	E_max_^a^ (S.E.)^b^	E_rate_	T_50_	R^2^
0	97.86 a (1.22)	0.8003	4.50	0.9673
1	94.94 a (0.06)	0.1977	4.78	0.9999
2	86.07 b (0.32)	0.1695	4.80	0.9972
4	62.25 c (0.21)	0.2686	5.73	0.9981
6	46.53 d (0.27)	1.0053	7.82	0.9962
8	7.14 e (2.45)	0.0110	8.66	1.0000

^a^ Means within a column followed by different letters indicate significant differences (P < 0.05) according to Tukey’s test.

^b^ standard error of the mean.

These results were similar to those for other *C*. *melo* varieties, where emergence also decreased strongly with increasing soil depth [15, 22, and 32]. Low emergence rates at deeper depths (> 8 cm) were observed in *C*. *melo* var. *dudaim* Naud., where the emergence rate was 11% at 9 cm burial depth and emergence did not occur between 12 and 15 cm depth [15].

Several factors affect seedling emergence, such as light, temperature, soil characteristics, and the physical limitations of the seedling. However, this study found that light was not a prerequisite for field muskmelon seed germination. The low emergence by deeper buried seeds may be due to hypoxia and reduced rates of gaseous diffusion in the soil [37], and small-seed species may have insufficient seed reserves to enable them to reach the soil surface [38]. The results of this study suggest that no-till farming practices or shallow tillage might increase field muskmelon emergence. Therefore, deep tillage, which buries the field muskmelon seeds at soil depths below 8 cm, may discourage the emergence of this weed and could be an option to reduce the effect of field muskmelon occurrence on crop yields.

## Conclusions

The results of this study suggest that field muskmelon can germinate and emerge under a wide range of environmental conditions. Seed germination of this species was not influenced by light, and it is able to germinate under highly saline conditions. Field muskmelon seeds are sensitive to osmotic potential, so action could be taken to reduce the osmotic potential at appropriate times to prevent seed germination. Additionally, it can germinate over a pH range of 4 to 10, which suggested that pH was not a limiting factor on further spread of this weed. The highest seedling emergence was observed in shallow soil and the lowest seedling emergence occurred at a depth of 8 cm. Deep plowing the soil may be a promising method to limit the spread of field muskmelon in agricultural systems.
